# Successful pulsed methylprednisolone and convalescent plasma treatment in a case of a renal transplant recipient with COVID-19 positive pneumonia: a case report

**DOI:** 10.11604/pamj.2021.38.273.28577

**Published:** 2021-03-16

**Authors:** Muharrem Bayrak, Kenan Çadirci

**Affiliations:** 1 Department of Internal Medicine, Erzurum Regional Training and Research Hospital, Health Sciences University, Erzurum, Turkey

**Keywords:** COVID-19, renal transplantation, methylprednisolone, convalescent plasma, case report

## Abstract

Coronavirus 2019 disease (COVID-19) is a deadly disease that was first seen in Wuhan, China, and primarily affects the respiratory system, but also has different systemic involvements. It has caused 89 million cases and 1.9 million deaths worldwide. COVID-19 positive renal transplant recipients have a higher mortality rate than COVID-19 patients in the normal population. There is no specific treatment and follow-up protocol for COVID-19 infection in transplant recipients. COVID-19 treatment and immunosuppressive therapy choices are controversial. Recently, pulse steroid therapies have been used in cases with severe COVID-19 pneumonia. Convalescent plasma therapy is used limitedly in COVID-19 patients. Our 49-year-old male patient has been a recipient of a renal transplant from a cadaver for 6 years. We aimed to make an additional contribution by presenting our patient to the literature whose COVID-19 PCR-RT test performed in the emergency department due to the complaints of fever, shortness of breath, and cough for five days was positive and had moderate COVID-19 pneumonia in thorax tomography and had serious clinical and radiological improvement after pulsed methylprednisolone and convalescent plasma therapy in the early period.

## Introduction

Coronavirus 2019 disease (COVID-19) is a deadly disease that was first seen in Wuhan, China, primarily involving the respiratory system with different systemic involvements [1,2]. It caused 89 million cases and 1.9 million deaths worldwide [3]. The risk of viral infection and mortality due to chronic immunosuppressive therapy in renal transplant recipients with COVID-19 infection is higher than in the normal population. The severity and duration of infection and prognosis may be worse due to immunosuppression [4,5]. There is no specialized treatment follow-up protocol in immunosuppressive therapy and infection treatment in COVID-19 renal transplant recipients [6]. Corticosteroid therapy is frequently used in patients hospitalized with COVID-19 infection. Recently, high-dose steroid treatments are used in patients with severe COVID-19 pneumonia [7,8]. Although there is no clear protocol for methylprednisolone treatment in the treatment of COVID-19 infection in renal transplant recipients, it is used effectively [9]. Although convalescent plasma therapy is a potentially effective treatment in the treatment of COVID-19, its difficulty in obtaining it and its use according to indications may cause problems for treatment. Convalescent plasma therapy was used in the treatment of COVID-19 infection successfully in the renal transplant recipient, using a donor who had previously had COVID-19 infection [10,11]. In our case, after the diagnosis of moderate COVID-19 pneumonia was made in the renal transplant recipient, we obtained a successful clinical and radiological response in our patient with severe clinical complaints with pulse methylprednisolone and convalescent plasma therapy in the early period. Since there is no clear follow-up and treatment protocol in the treatment of COVID-19 in renal transplant recipients, we think that we contribute to the literature by presenting our experience in our case.

## Patient and observation

A 50-year-old male patient was followed up for 14 years with a diagnosis of chronic glomerulonephritis. He had hemodialysis for 8 years as a result of end-stage renal failure due to this disease. A renal transplant operation with a kidney taken from a cadaver donor has been performed for the last 6 years. Serological tests for hepatitis B, hepatitis C, human immunodeficiency virus (HIV), cytomegalovirus (CMV), Epstein-Barr virus (EBV), varicella-zoster virus (VZV) before transplant were reported as negative. Our case had type 2 diabetes mellitus for 5 years and hypertension for 9 years.

**Figure 1:**
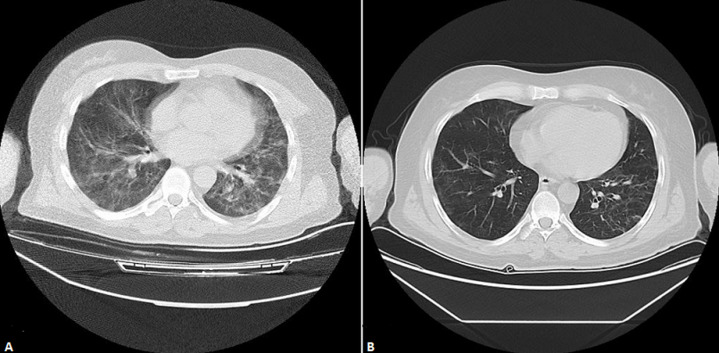
thoracic tomography findings before and after treatment

He was using medication as mycophenolate mofetil (500 mg/day), cyclosporine (100 mg/day), prednisolone (5mg/day), insulin glargine 18 units, acetylsalicylic acid (100mg/day), pantoprazole (40 mg/day), valsartan 50 mg/day. Fever that started in the last five days was 38.6°C, pulse 98/min, respiratory rate 24/min, blood pressure 142/95 mm Hg, and oxygen saturation was measured by pulse oximetry 88%. On physical examination, there were rales in respiratory sounds in the lower lobes of both lungs. In our case, it is seen that the COVID-19 PCR-RT test performed as a result of these complaints is positive, and thoracic tomography shows consolidated areas of peripherally located ground glass density and crazy paving findings, which are evident in the lower lobe basal segments in both lungs (Figure 1). He was admitted to the infection service because of moderate COVID-19 pneumonia on thoracic tomography. Laboratory findings on the first day in the service were hemoglobin 14.8 g/dL (14.1-17.8), white blood cell (WBC) 7.2x10^3^ (3.91-10.9 x10^3^), platelet (PLT) 101x10^3^ (152-383x10^3^), neutrophil 62.2% (40-74%), lymphocyte count 0.42 x10^3^ μL (1.21-3.77x10^3^ μL), serum creatine: 1.36 (0.7-1.7 mg/dl), urea: 36 (9-23 mg/dl), glomerular filtration rate (GFR) 60.6 ml/min C-reactive protein (CRP) 116.2 mg/L (0-5 mg / L), D-dimer 1544 mgL (0-500 mgL), procalcitonin 0.04 ng/ml (0-0.05 ng/ml), fibrinogen 224 mg/dL (200-400 mg/dL), ferritin 1650 ng/ml (22-232 ng/ml), AST 55 IU/L (0-40 IU/L), ALT 65 U/L (7-40 U/L), lactate dehydrogenase (LDH) 594 U/L (230-500 U/L), albumin 4.2 mg/dl (3.2-4.8 g/dl), glycated hemoglobin level (HbA1c) 9.1, cyclosporine blood level 88 ng/ml, arterial blood gas: pH: 7.39, PO_2_2 88.4 PCO_2_: 34.1 HCO_3_23.2 BE: -2 SpO_2_: 88. H1N1 and other viral serological markers were negative. Mycophenolate mofetil and prednol were discontinued, and cyclosporine was started to be given at half dose. Favipiravir 2x1600 mg loading and 2x600 mg maintenance (10 days), 3-4 lt/min oxygen therapy with a nasal cannula, subcutaneous 40 mg/day enoxaparin, acetyl cysteine 900 mg/day, meropenem 1 g every 12 hours, intravenous treatment was initiated. Since our patient had high CRP values, low oxygen saturation, and moderate involvement in thoracic tomography, methylprednisolone 250 mg/day, a 1-hour intravenous infusion was given for 3 days. After 3 days of pulse methylprednisolone treatment, 40 mg/day methylprednisolone maintenance treatment was continued in our patient.

**Figure 2:**
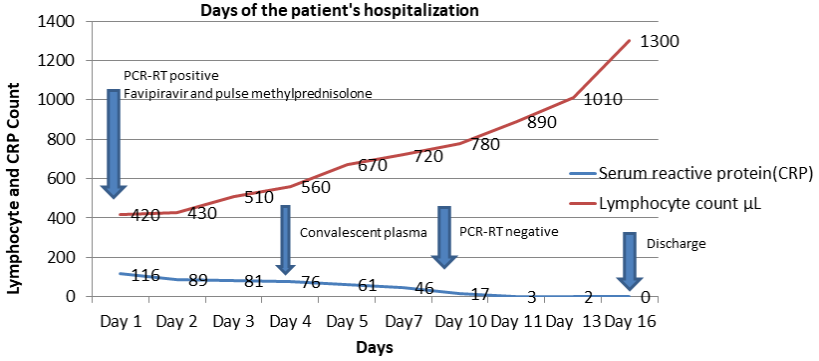
treatment process during the days of the patient's hospitalization

Our patient's CRP and lymphocyte count were followed up (Figure 2). On the 4^th^ day in the hospital, 200 ml of convalescent plasma taken from a donor with a previous COVID-19 infection, and a sufficient antibody level was given intravenously for 3 days. No complications occurred during convalescent plasma and pulse steroid therapy. Blood, sugar, and blood pressure arterioles were monitored, intensive insulin therapy was initiated. In our case, no growth was detected in blood, urine, and throat cultures. On the 10^th^ day of the treatment of our patient, antiviral treatment was discontinued, methylprednisolone treatment was reduced to 20 mg/day. In the control thoracic tomography, a minimal ground-glass opacity is observed in the lower lobes of both lungs (Figure 1). In the laboratory, HB: 14.2 g/dL, WBC 8.3x10^3^, PLT 202x10^3^, neutrophil 58.4%, lymphocyte count 0.78 x10^3^μL, serum creatine: 1.04, urea: 21, GFR 83.9. ml/min, CRP 7.1 mg/L, D-dimer 614 mgL, procalcitonin 0.06 ng/ml, ferritin 1004 ng/ml, oxygen saturation 98%. As a result of clinical and radiological improvement in the clinical follow-up of our case, normalization of vital signs, and improvement of oxygen saturation, the treatment before admission to the service was restarted, and he was discharged with subcutaneous 40 mg/day enoxaparin added as anticoagulant treatment.

## Discussion

The incidence of COVID-19 infection in renal transplant recipients is gradually increasing. The T cells immune system is suppressed due to the long-term immunosuppressive agents used by renal transplant recipients. In some case series, acute renal injury and mortality rates have been observed more frequently than in the general population [12]. Our case had similar symptoms, such as cough, shortness of breath, and fever, similar to the literature [13]. There are no proven algorithms in antiviral and immunosuppressive treatment management in COVID-19 infection in renal transplant recipients. Oseltamivir, remdesivir, hydroxychloroquine, lopinavir/ritonavir, and favipiravir treatments were used in studies in COVID-19 positive renal transplant recipients. No definitive efficacy of antiviral treatments has been proven in the treatment of COVID-19. Favipiravir treatment was used in our case and no side effects were observed during the treatment process. Mycophenolate mofetil treatment was discontinued until the viral infection was controlled, cyclosporine dose was halved. There is no proven protocol in the literature regarding the immunosuppressive agents used by renal transplant recipients during the treatment of COVID-19 infection. It is known that mycophenolate mofetil increases the progression in viral infections because of inhibiting T and B lymphocyte activation and proliferation, whereas cyclosporine does not have such an effect on T lymphocytes [14].

Mycophenolate mofetil treatment was discontinued to prevent the progression of COVID-19 viral pneumonia. The frequency of bacterial pneumonia due to chronic immunosuppression has increased in renal transplant recipients, therefore, meropenem, a broad-spectrum antibiotic, was chosen for the initial treatment of our case [15]. In the treatment of COVID-19 infection in renal transplant recipients, steroid therapy is used for treatment to prevent inflammation and adrenal insufficiency. Methylprednisolone treatment has been used at different doses in case series [16]. Pulse methylprednisolone therapy is an outstanding treatment option in patients with COVID-19 infection in severe clinical cases, cytokine storm, and macrophage-activation syndrome. In our case, because of hyperinflammation due to high CRP values, decreased oxygen saturation, moderate viral pneumonia on thoracic tomography, lymphopenia, high D-dimer and ferritin, pulsed methylprednisolone treatment was applied as aggressive treatment. Ruiz-Irastorza *et al*. [17] successfully applied pulse methylprednisolone therapy in the second week of treatment in a patient with COVID-19 positive renal transplant recipient due to high CRP values and severe viral pneumonia. In our case, unlike this case, the existing hyperinflammation was prevented by applying pulse steroid therapy in the early period. Also, we think that the cytokine storm, severe lung damage, and the need for intensive care in our patient were prevented by early treatment. Sauñe PM *et al*. [8] applied 500 mg/day 3-day pulse methylprednisolone treatment in the renal transplant recipient patient due to cytokine storm in the late period after the cytokine storm developed and a positive clinical response was obtained. In our case, clinical deterioration was prevented by applying it in an earlier period.

Convalescent plasma therapy has limited use in COVID-19 patients due to the difficulty in finding ABO-compatible plasma and various contraindications. In the COVID-19 case series in China, CRP decreases in the early period and clinical improvement in oxygen saturation were observed in patients who received convalescent plasma [18,19]. In the study of Jiang J *et al*. [11], a 70-year-old patient with a poor prognosis who has been a recipient of a renal transplant for 10 years had positive clinical results. Our case is a younger patient. Although the CRP values were higher, oxygen saturation was lower, and in our case, using high-dose methylprednisolone and administering convalescent plasma for 3 days made the difference between the two cases. In both cases, the 4^th^ day of treatment and the early use were similar.

## Conclusion

We applied pulse methylprednisolone and convalescent treatment as an aggressive treatment due to moderate COVID-19 pneumonia, high CRP, D-dimer, ferritin level in the renal transplant recipient candidate and since there is no contraindication in the early period before complications as cytokine storm, macrophage activation syndrome and acute respiratory stress syndrome developed in the patient. Renal transplant recipient patients have unproven and different treatment options for COVID-19 infection. We aimed to make an additional contribution to the current literature considering that the early stage methylprednisolone and convalescent plasma treatment we used in our case could be an outstanding treatment in this regard.

## References

[1] Ning L, Liu L, Li W, Liu H, Wang J, Yao Z (2020). Novel coronavirus (SARS-CoV-2) infection in a renal transplant recipient: case report. Am J Transplant.

[2] Dirim AB, Demir E, Ucar AR, Garayeva N, Safak S, Oto OA (2020). Fatal SARS-CoV-2 infection in a renal transplant recipient. CEN Case Rep.

[3] Hopkins J (2020). Johns Hopkins Coronavirus Resource Center. COVID-19 Case Tracker.

[4] Hilbrands LB, Duivenvoorden R, Vart P, Franssen CF, Hemmelder MH, Jager KJ (2020). COVID-19-related mortality in kidney transplant and dialysis patients: results of the ERACODA collaboration. Nephrol Dial Transplant.

[5] Zhu L, Gong N, Liu B, Lu X, Chen D, Chen S (2020). Coronavirus disease 2019 pneumonia in immunosuppressed renal transplant recipients: a summary of 10 confirmed cases in Wuhan, China. Eur Urol.

[6] Tanaka R, Kakuta Y, Tsutahara K, Nakagawa M, Ichimaru N, Sakaguchi K (2020). Successful recovery from coronavirus disease 2019 in a living kidney transplant recipient using low-dose methylprednisolone. IJU case reports.

[7] Chen Q, Song Y, Wang L, Zhang Y, Han L, Liu J (2021). Corticosteroids treatment in severe patients with COVID-19: a propensity score matching study. Expert Rev Respir Med.

[8] Sauñe PM, Bryce-Alberti M, Portmann-Baracco AS, Accinelli RA (2020). Methylprednisolone pulse therapy: an alternative management of severe COVID-19. Respiratory Medicine Case Reports.

[9] Zhu L, Xu X, Ma K, Yang J, Guan H, Chen S (2020). Successful recovery of COVID-19 pneumonia in a renal transplant recipient with long-term immunosuppression. Am J Transplant.

[10] Bloch EM, Shoham S, Casadevall A, Sachais BS, Shaz B, Winters JL (2020). Deployment of convalescent plasma for the prevention and treatment of COVID-19. J Clin Invest.

[11] Jiang J, Miao Y, Zhao Y, Lu X, Zhou P, Zhou X (2020). Convalescent plasma therapy: helpful treatment of COVID-19 in a kidney transplant recipient presenting with severe clinical manifestations and complex complications. Clin Transplant.

[12] Akalin E, Azzi Y, Bartash R, Seethamraju H, Parides M, Hemmige V (2020). COVID-19 and kidney transplantation. N Engl J Med.

[13] Devresse A, Belkhir L, Vo B, Ghaye B, Scohy A, Kabamba B (2020). COVID-19 infection in kidney transplant recipients: a single-center case series of 22 cases from Belgium. Kidney Med.

[14] Allison AC, Eugui EM (1994). Preferential suppression of lymphocyte proliferation by mycophenolic acid and predicted long-term effects of mycophenolate mofetil in transplantation. Transplant Proc.

[15] Chang GC, Wu CL, Pan SH, Yang TY, Chin CS, Yang YC (2004). The diagnosis of pneumonia in renal transplant recipients using invasive and noninvasive procedures. Chest.

[16] Viana LA, Cristelli MP, Ficher KN, Rezende JT, Villanueva LA, Santos DW (2021). Kidney transplantation in patients with SARS-CoV-2 infection: a case series report. Transplantation.

[17] Ruiz-Irastorza G, Pijoan JI, Bereciartua E, Dunder S, Dominguez J, Garcia-Escudero P (2020). Second week methyl-prednisolone pulses improve prognosis in patients with severe coronavirus disease 2019 pneumonia: an observational comparative study using routine care data. PloS one.

[18] Duan K, Liu B, Li C, Zhang H, Yu T, Qu J (2020). The feasibility of convalescent plasma therapy in severe COVID-19 patients: a pilot study. MedRxiv.

[19] Chen L, Xiong J, Bao L, Shi Y (2020). Convalescent plasma as a potential therapy for COVID-19. Lancet Infect Dis.

